# Simultaneous recording of electrical and metabolic activity of cardiac cells *in vitro* using an organic charge modulated field effect transistor array

**DOI:** 10.3389/fbioe.2022.945575

**Published:** 2022-08-04

**Authors:** Andrea Spanu, Laura Martines, Mariateresa Tedesco, Sergio Martinoia, Annalisa Bonfiglio

**Affiliations:** ^1^ Department of Electrical and Electronic Engineering, University of Cagliari, Cagliari, Italy; ^2^ Department of Informatics, Bioengineering, Robotics and Systems Engineering, University of Genova, Genova, Italy; ^3^ Interdepartmental Center for Amyotrophic Lateral Sclerosis and Motor Neuron Diseases, Cagliari, Italy; ^4^ Scuola Universitaria Superiore IUSS, Pavia, Italy

**Keywords:** OCMFET Array, *in vitro* cardiomyocytes cultures, metabolic activity, electrophysiological activity, simultaneous multiparametric monitoring

## Abstract

*In vitro* electrogenic cells monitoring is an important objective in several scientific and technological fields, such as electrophysiology, pharmacology and brain machine interfaces, and can represent an interesting opportunity in other translational medicine applications. One of the key aspects of cellular cultures is the complexity of their behavior, due to the different kinds of bio-related signals, both chemical and electrical, that characterize these systems. In order to fully understand and exploit this extraordinary complexity, specific devices and tools are needed. However, at the moment this important scientific field is characterized by the lack of easy-to-use, low-cost devices for the sensing of multiple cellular parameters. To the aim of providing a simple and integrated approach for the study of *in vitro* electrogenic cultures, we present here a new solution for the monitoring of both the electrical and the metabolic cellular activity. In particular, we show here how a particular device called Micro Organic Charge Modulated Array (MOA) can be conveniently engineered and then used to simultaneously record the complete cell activity using the same device architecture. The system has been tested using primary cardiac rat myocytes and allowed to detect the metabolic and electrical variations thar occur upon the administration of different drugs. This first example could lay the basis for the development of a new generation of multi-sensing tools that can help to efficiently probe the multifaceted *in vitro* environment.

## 1 Introduction

Research involving *in vitro* cell cultures often relies on the monitoring of cellular metabolism as the main parameter to get real time information on the culture’s health, as well as to investigate specific cellular responses to external stimuli (e.g. electrical and pharmacological). As recently reported by Kieninger and colleagues (Kieninger et al., 2018), understanding the metabolic activity of a cellular assembly is essential in fundamental research but also in other practical applications such as pharmacology, as the reproducibility of the experimental trials is often conditioned by the metabolic state of the culture under test. Moreover, there are other applications such as drug testing, in which metabolic activity can provide useful information on the interaction between the cells and specific molecules under test, as well as on the overall biocompatibility of a chemical compound. Given the paramount importance of being able to assess the cellular metabolic activity during an *in vitro* experiment, several different approaches have been investigated and used during the past 40 years. In particular, metabolic activity can be indirectly measured by analysing parameters like oxygen uptake (due to cellular respiration) (Wu et al., 2010; Kieninger et al., 2014), lactate production (due to anaerobic processes) and glucose consumption (during both aerobic and anaerobic conditions) (Boero et al., 2011; Koman et al., 2015), and variations of the pH of the extracellular medium (Baumann et al., 1999; Lorenzelli et al., 2003; Munoz-Berbel et al., 2013; McBeth et al., 2018; Sakata et al., 2018; Dantism et al., 2019).

Within the realm of cellular experimentation, excitable cells assemblies occupy a prominent role. In fact, the capability of electrogenic cells such as muscle and neuronal cells to communicate through voltage difference-based electrochemical signals, has attracted a lot of interest in the research community since the beginning of modern science. In particular, during the last 70 years, there has been an impressive and steadily growing effort on the development of algorithms for the extraction of the information from cellular spiking activity, both *in vitro* and *in vivo* (Maccione et al., 2009; Pani et al., 2011; Carta et al., 2013), and increasingly complex *in vitro* cellular models, thanks to the availability of Micro Electrodes Array (MEA) systems and CMOS high-density devices, capable of interfacing both standard, planar cellular cultures and 3D cellular assemblies (Weis et al., 1996; Chiappalone et al., 2006; Berdondini et al., 2009; Frey et al., 2010; Ballini et al., 2014; Dragas et al., 2017; Lorenzelli et al., 2019; Brofiga et al., 2020; Spanu et al., 2020; Muzzi et al., 2021; Xu et al., 2021a).

Having the possibility of simultaneously recording both metabolic activity and electrophysiological signals within a cellular culture could offer a more complete description of the fundamental mechanisms that drive its behaviour, thus allowing to better understand the phenomena (both physiological or pathological) in which those cells are involved. However, despite some interesting approaches (Wu et al., 2013; Pemberton et al., 2014; Song et al., 2021), a definitive solution for the simultaneous monitoring of both kind of signals is yet to be proposed. A possible interesting way to approach the problem has recently risen from the fervent field of organic bioelectronics (Rivnay et al., 2014; Torricelli et al., 2021). In fact, in the last 2 decades, the fast growth of organic electronics, and in particular of organic transistor-based sensors and biosensors, has led to the development of a new generation of measuring devices for cellular applications, including those for electrophysiological and metabolic activity monitoring, as recently reported in (Spanu et al., 2021a). This new technology brings about several advantages with respect to the previously mentioned approaches, like the reduction of production costs and the possibility to employ materials such as plastic or paper, thus making it possible to design flexible devices with large area fabrication methods (Xu et al., 2021b). In particular, there have been recently some attempts of using mainly Organic Electrochemical Transistors (OECTs) for the *in vitro* detection of the electrical (Gu et al., 2019) and metabolic (Pappa et al., 2018; Moldero et al., 2020; Cacocciola et al., 2022). However, the recent scene is characterized by a lack of systems, based on organic tools or standard silicon-based devices, that can effectively provide multi-sensing capabilities in a convenient yet reliable way.

In this work, we present a flexible, low-cost and highly sensitive device, called MOA or Micro OCMFET Array with up to 56 recording sites, specifically designed to simultaneously *in vitro* monitor both the electrophysiological activity of cells and the pH variations of the culture medium, thus providing indirect information about their metabolism. The device is based on a peculiar organic field effect transistor (OFET) structure called OCMFET (Organic Charge Modulated Field Effect Transistor), a versatile organic sensing device that can be selectively functionalized in order to become sensitive to different parameters, ranging from DNA sensing to temperature and force sensing (Napoli et al., 2018; Viola et al., 2018; Pattison et al., 2019), and that recently demonstrated its potential in the field of *in vitro* electrophysiology (Spanu et al., 2015; Spanu et al., 2018). The OCMFET proved to be a very interesting device for cellular applications due to an unprecedented charge sensitivity, the absence of a reference electrode (thanks to the presence of a second gate through which is possible to set the transistor’s working point), and an elongated gate structure that allows to selectively functionalize the sensing area while at the same time helping to maximize the stability of the organic semiconductor. We here report the preliminary results obtained using a MOA device with *in vitro* cultures of primary cardiac rat cardiomyocytes, whose activity (both metabolic and electrical) has been ad hoc modulated by pharmacological stimulation. The preliminary results highlight the possibility of deriving meaningful information about the state of a cell culture by the simultaneous recording the electrophysiological and metabolic activities and with a low-cost, easy-to-use integrated approach, thus demonstrating for the first time the feasibility of using this organic platform for multi-sensing *in vitro* monitoring applications.

## 2 Materials and methods

### 2.1 Device fabrication

The devices employed in this paper were fabricated onto a 250 μm-thick polyethylene terephthalate (PET) substrate and are constituted by an array of 16 OCMFETs, two of which are specifically functionalised to monitor the extracellular pH variations. The floating gates of the OCMFETs are made of aluminum, with an additional titanium layer deposited only over the sensing areas to ensure a biocompatible and stable interface with the cells.

**FIGURE 1:**
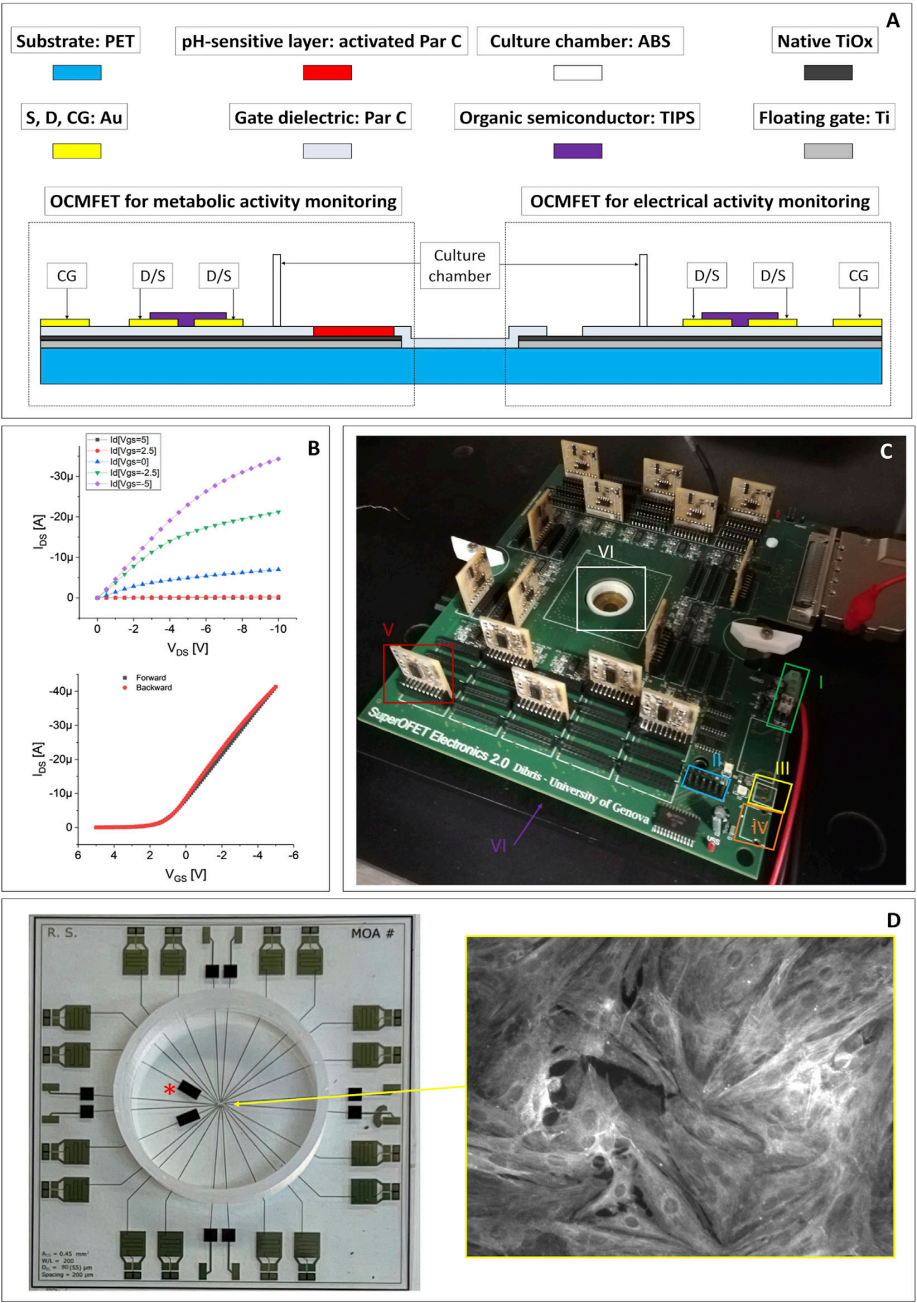
**(A)** Cross section and materials of a MOA for the simultaneous *in vitro* recording of the electrical and the metabolic activity of cell cultures (drawing not to scale). **(B)** Representative electrical output (top) and input (bottom) characteristics of one of the OCMFET employed in the experiments. **(C)** The readout electronics employed for the experiments: 1) dual stabilized external power supply; 2) connector for the programming of the digital potentiometers used to set each transistor’s working point; 3) USB external power supply; 4) single not stabilized external power supply; 5) modular PCBs (one for each channel), with the amplification of the output of each transistor (the I/V conversion is performed on the main board, right next to the recording site); 6) culturing chamber. **(D)** Top view of a completed MOA with the pH channels (*) and a cardiomyocytes culture fixed and then immunostained for the sarcomeric α-actinin protein (inset).

The sensing areas (i.e. the extended parts of the floating gates) are differently processed according to the specific sensing requirements of the devices. For the devices dedicated to monitor the electrical activity of cells, a 150 nm-thick Parylene C layer is deposited by Chemical Vapor Deposition on top of the floating gate. After the deposition, a selective removal of Parylene C is done by oxygen plasma etching in order to expose a specific portion of the floating gate to the direct contact with cells. As for the pH-sensitive devices a 1 μm-thick layer of Parylene C is deposited followed by a selective plasma activation (which, in addition of reducing its thickness to 500 nm, also causes the superficial exposure of carboxyl groups that can be protonated or de-protonated according to the pH of the surrounding medium), in order to turn it into an ultra-sensitive, and stable pH sensor with a supernernstian response up to more than 1 V/pH, as recently demonstrated (Spanu et al., 2017). The following steps are the deposition and the patterning of gold sources, drains and control gates, and the deposition of the organic semiconductor (i.e. TIPS-pentacene) by drop casting. A more detailed description of the fabrication protocol can be found in a recent publication from this group (Spanu and Annalisa, 2021b). The cross section of a MOA device for the simultaneous recording of cellular metabolic and electrical activity is shown in Figure 1A. The sensing areas for electrophysiological activity have a radius of 50 μm, and a total surface of 0.31 mm^2^ and are distant (from centre to centre) 380 μm, while the sensing areas for pH monitoring have an area of 4.5 mm^2^. In Figure 1B, the output and input characteristics of one OCMFET from one MOA are shown.

### 2.2 Experimental setup

The experimental setup is composed by a modular, custom readout electronics with a variable number of channels (up to 56) and adaptable filtering stages, in order to be versatile and easily adaptable to different kinds of sensors (Figure 1C). In particular, the first stage for the I/V conversion is placed in the motherboard as close as possible to the transistors in order to reduce the noise, while filters and conditioning stages are situated in secondary PCBs, connected to the main one through DF3 connectors so that they can be modified and easily replaced to respond to the specific experimental needs. In the same secondary PCBs are situated also the polarizing systems, to independently operate each OFET. The gate voltage (VGS) and drain voltage (VDS) of each transistor are automatically adjusted by setting the desired output current thanks to programmable digital trimmers and a simple Arduino routine.

In the proposed work, we set the number of monitored channels to 16, the filters of 14 of which are designed to record the electrophysiological activity, while the other two have different stages, conceived to monitor the pH variations. Both channels are characterized by an I/V converter with a feedback resistance of 1MΩ; in the case of the electrophysiological signals the filtering block is a bandpass filter composed by two Butterworth filters: a fourth order high pass and a second order low pass, to obtain a bandwidth of 150 Hz-3 kHz, with a voltage gain of 100. The filtering stage for the pH sensors is composed by a low pass Butterworth filter of eighth order with a cut-off frequency of 10 Hz, with unitary gain. The described electronics has been designed to fit on a commercial MultiChannel System ground plate, to exploit its integrated heater; in addition, the electronics is connected to a MultiChannel System acquisition board that perform the A/D conversion, the acquisition, and the storage of data. All the measurement sessions were carried out inside a Faraday cage in order to minimize the electrical environmental noise on the system. The metabolic trace has been down-sampled to 1 kHz using a decimation procedure (from the initial 10 kHz), to remove the high frequency artifacts induced by the manual introduction of the drugs. The Mean Firing Rate (MFR) is calculated starting from the electrophysiological signal and represents the number of spikes in 1 minute of recording. It is worth underlining here that no external reference electrode is needed to operate the device, allowing a compact integration of the system components on the same surface and the possibility of separately addressing each transistor of the array.

### 2.3 Cell culture

Cell suspension of cryopreserved post-natal rat’s cardiomyocytes (QBM Cell science R-CM-561), after being thawed in a water bath a 37°C for 3 min, was gently transferred in a sterile centrifuge tube with DMEM:M199 plating medium. The conical tube was then swirled during the addition of another 1 ml of the medium. Then, a small sample of cell suspension has been removed to perform a cell count using hemocytometer and Trypan blue to identify viable cells. Cardiomyocytes were centrifuged at 180 x g for 5 min and resuspended in the necessary amount of the plating medium (DMEM: M199 4:1, HS 6%, FBS 4%, Glutamax 1% 10 μg/ml Gentamycin) to achieve the desired density of 800 cells/mm^2^. After that, cardiomyocytes are finally plated onto the MOA’s sensing area in about 200 μL of solution and the culture is placed in a cell culture incubator at 37°C, 5% CO_2_. A few hours after plating, the total volume of culture medium is increased to 500 μl and after 48 h the medium is carefully aspirated to wash out non-adherent cells while the plating medium is substituted with a maintenance medium composed by Neurobasal, β27, 1% glutamax, 1% horse serum and 25 μg/ml Gentamycin. Starting from the third day, the horse serum component has been omitted. From then on, the culture medium has been changed every 2 days (Becerra et al., 2021). In Figures 1A,D healthy culture of primary rat cardiomyocytes immuno-stained for the sarcomeric α-actinin protein is shown. The day before cell plating, the culture chamber is sterilized through a mild oxygen plasma followed by a wash with 70% ethanol solution. The culture chamber is then rinsed with sterile water prior the coating with adhesion factor (laminin, 50/80 μg/ml), and eventually the devices are stored into the incubator for 12 h. Finally, the day of the cells collection, the solution containing the adhesion factor is removed and the chamber rinsed with 300 µl of sterile water.

## 3 Results and discussion

The choice of using commercially available primary cardiac cells allowed us an easy assessment of their viability (using a simple optical inspection for the evaluation of their contractile activity), and at the same time offered a relatively simple way to demonstrate the potential of the proposed approach, thanks to their well-known pharmacological response to commercial drugs with inotropic and chronotropic effects. The pharmacological experiments have been carried out 5 days after the cells plating. Approximately 20–30 min before starting the recordings, the culture medium has been replaced with a 300 μl of low-buffered Tyrode solution, in order to be able to appreciate the small local pH variations of the culture medium with the pH-sensitive OCMFETs. Thereafter, the cultures have been stored again into the incubator. The goal of the experiment was to observe the effect of two specific drugs on the electrical and metabolic signals simultaneously recorded by means of the proposed system. The cardiomyocytes’ activity has been modulated using increasing doses of the two drugs, namely Isoprenaline, a non-selective *ß* adrenoreceptor agonist that has positive inotropic and chronotropic effects, and Verapamil, a calcium blocker that acts as a cardio-relaxant (Pointon et al., 2015).

**FIGURE 2:**
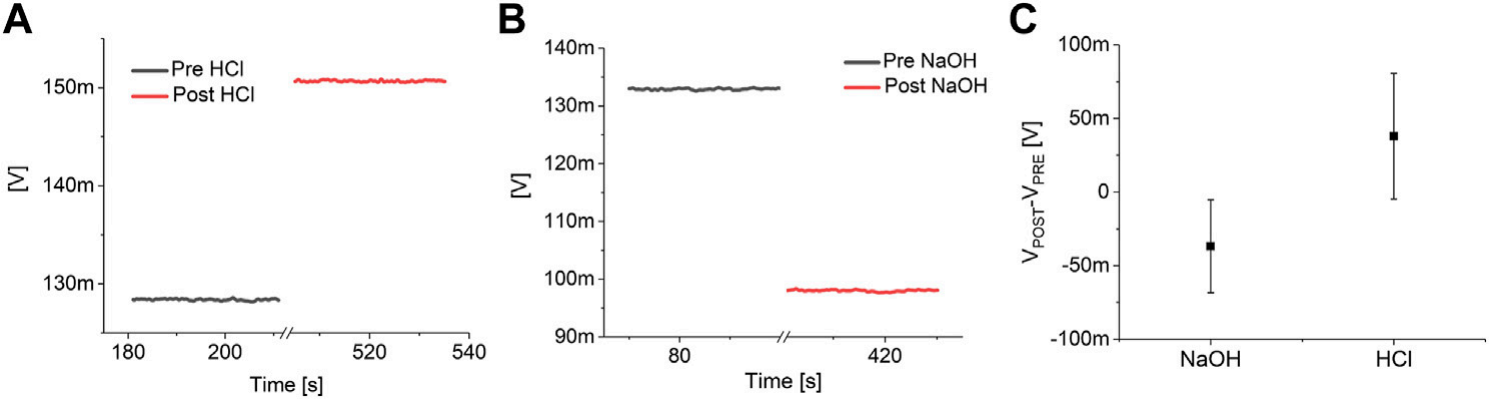
pH response analysis. Example of the response of a pH channel of a MOA without the cells to a strong acidification **(A)** and basification **(B)**. **(C)** Analysis on the effect of a strong acidification (pH 7.3 to pH 1) and basification (pH 7.3 to pH 13) on six blanks (three blanks each).

**FIGURE 3:**
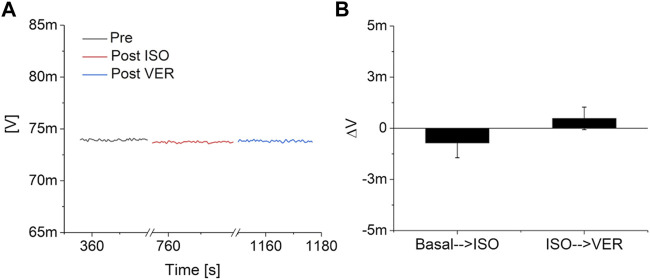
Drugs response analysis. **(A)** Example of the response of a pH channel of a MOA without the cells to the consecutive addition of 10 nM of Isoprenaline (ISO) and 40 μM Verapamil (VER). **(B)** Analysis on three blanks of the effect of the two drugs on the output of the system.

The response of the devices to solutions with different pH and to Isoprenaline and Verapamil have been preliminary evaluated by an initial characterization on nine blanks (i.e. devices measured in the exact experimental conditions but without the cells), six for pH characterization and three for drug response. In particular, three blanks were exposed to a strong acidification (from pH 7.3 to pH 1) by adding hydrochloric acid (final concentration: 0.1 M) to the same lowly buffered Tyrode’s solution, three blanks were exposed to a strong basification (from pH 7.3 to pH 13) using sodium hydroxide (final concentration: 0.1 M), and three more blanks were tested in a simulated experimental session, with the addition of Isoprenaline and Verapamil. The preliminary tests highlighted a consistent increase in the output signal during acidification and a reduction during basification for all the tested devices (Figure 2). On the contrary, no significant variations of the output of the blanks have been observed upon the addition of 10 nM of Isoprenaline and 40 μM of Verapamil (Figure 3). These preliminary experiments allowed to make sure that the variations observed on the device output during the pharmacological experiments are indeed the effect of the cell activity (both metabolic and electrical).

**FIGURE 4:**
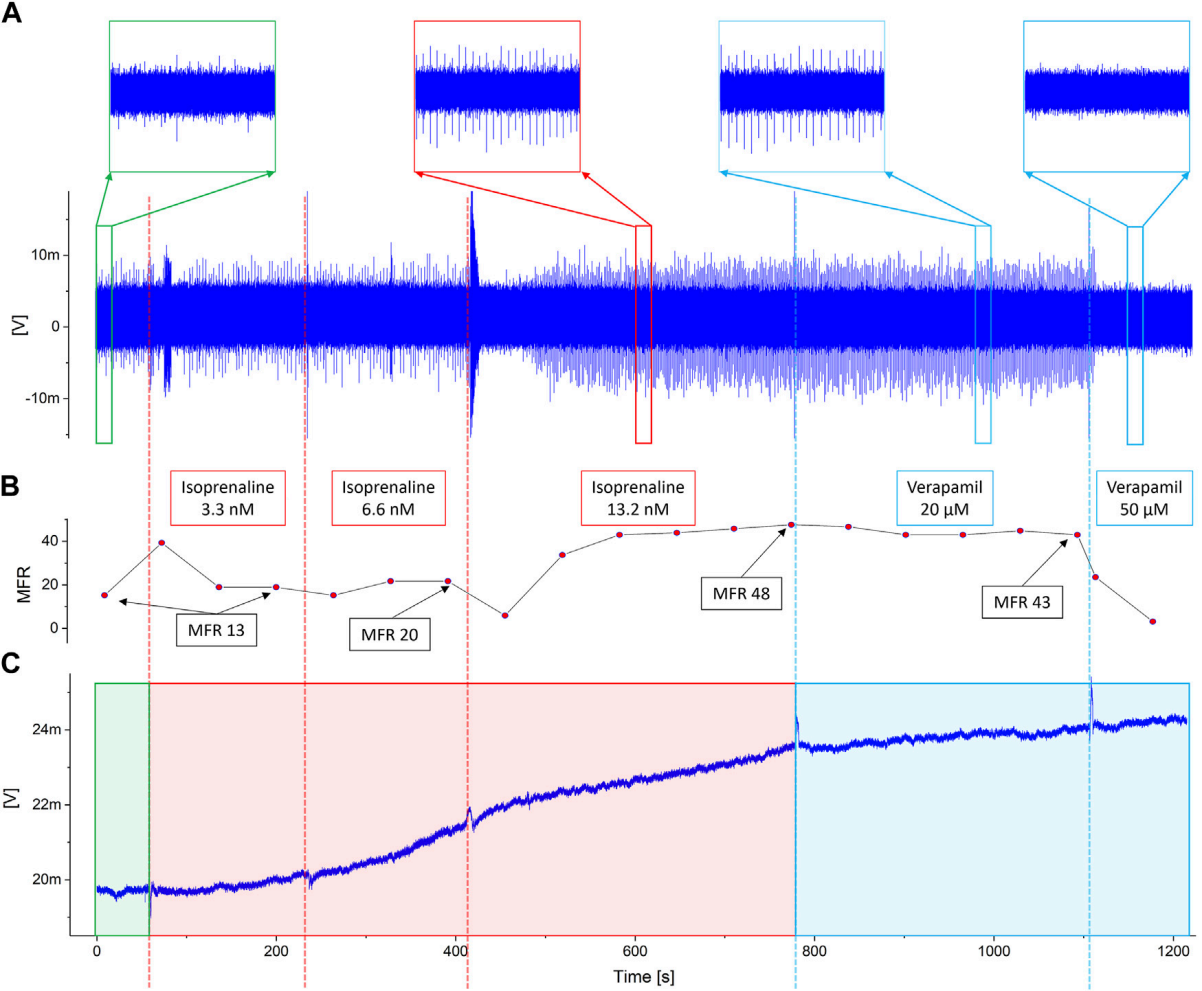
Experimental results. The dotted vertical lines represent the timestamps of the addition of Isoprenaline (red) and Verapamil (blue). **(A)** The output of the channel for cellular electrical activity shows how noticeable variations occur only when the maximumdoses of drugs have been reached. This is also confirmed observing the inset, in which detailed views of the signal during the basal (green square), the highest dose of isoprenaline (red square) and Verapamil (blue squares) are shown. **(B)** Mean Firing Rate during the whole pharmacological experiment. The labels specify the MFR value at the end of each phase. **(C)** Output of the pH-sensitive channel. Interestingly, noticeable changes in the activity can be appreciated right after the administration of the lower doses of both isoprenaline and verapamil.

The experimental results are shown in Figure 4. In particular, Figure 4A shows the output signal of an OCMFET for cellular electrical activity, Figure 4B shows the mean firing rate during the whole pharmacological experiment, and Figure 4C shows the simultaneously recorded output of an OCMFET for the cellular metabolic activity (i.e. pH variations). By analysing the electrophysiological data alone, the effect of the lowest doses of either Isoprenaline and Verapamil is not clearly observable. Indeed, the spiking frequency seems to considerably change only when higher doses (>6.6 nM) are administered to the culture, as clearly noticeable from the mean firing rate, being this result in line with previous studies (Reppel et al., 2007). Interestingly, by looking at the metabolic trace, it is possible to derive some qualitative, though meaningful, additional information, as the effect of the two drugs is clearly appreciable in terms of the induced changes of the rate at which the output of the pH-sensitive transistor drifts as a consequence of the change in the extracellular acidification rate. In particular, when Isoprenaline is added to the solution, an increase of the output’s drift can be appreciated (from flat to 0.3 mV/min), an effect that is partially reversed after the addition of Verapamil, with a reduction of the drift (from 0.3 mV/min to 0.07 mV/min). The slight drift in the basal phase has been subtracted in order to highlight the effect of the addition of the two drugs. Interestingly, these effects can be appreciated already at the lowest doses of either drugs (respectively 3.3 nM and 20 μM), well in advance if compared to the relative changes in the spiking frequencies of the culture. In fact, while the spiking frequency increases only slightly during the first two phases in which Isoprenaline is added, the metabolic activity shows a clear variation of the curve slope right after the first administration. On the other hand, when Verapamil is added to the solution, the slope visibly changes in the opposite direction right after the first injection of the calcium blocker compound, thus indicating a possible early effect on the culture’s activity. Interestingly, Verapamil is well-known for its immediate effect (in the 10-6-10-5 M range) on the contractile ability of cardiac cells (McCall, 1976), due to its strong and very rapid inhibitory effect on the Na influx. On the contrary, Verapamil’s negative chronotropic effect on the field potentials frequency can in fact take up to several minutes (depending on the concentration), as demonstrated by Rappel et al. (Reppel et al., 2007). These considerations can of course vary with respect to cells density and cell types. Nevertheless, these preliminary results give a clear indication on the complexity and nuances of the system under test, and highlight the interesting potential of the proposed approach, which can ultimately provide deeper insight on the complex behavior of cell cultures.

## 4 Conclusion

We here showed the first example of the simultaneous recording of the electrical and the metabolic activity of a cell culture using a specialized organic transistor-based device called Micro OCMFET Array (MOA). The preliminary validation of the system with cardiac cultures demonstrated the possibility to get diversified and meaningful information about the change in the behavior of the culture upon the addition of different drugs by looking simultaneously at these two different key parameters, a feature that can be of great interest during *in vitro* cellular applications. In particular, a visible change in the acidification rate of the extracellular medium (indicated by a sudden change in the drift of the output current of the pH sensitive device) can be appreciated even for low doses of the drug, thus well before any visible change in the firing rate, which can be induced only by increasing the dose. These results, although preliminary, demonstrate the potential of such approach, that could also be enriched by recording other parameters of interest (for instance oxygen uptake or lactate concentration) by a suitable functionalization of the sensing area of the devices, another feature that is possible due to the peculiar structure of the OCMFET and the innovative MOA’s design. Thus, thanks to a simple fabrication process, a versatile organic charge sensor, and low-cost, biocompatible and flexible materials, this approach may lay the foundation for a new generation of *in vitro* tools with multi-sensing capabilities for electrophysiology and pharmacology applications.

## Data Availability

The raw data supporting the conclusions of this article will be made available by the authors upon request.
